# Synthesising results of meta-analyses to inform policy: a comparison of fast-track methods

**DOI:** 10.1186/s13750-023-00309-y

**Published:** 2023-08-21

**Authors:** David Makowski, Rui Catarino, Mathilde Chen, Simona Bosco, Ana Montero-Castaño, Marta Pérez-Soba, Andrea Schievano, Giovanni Tamburini

**Affiliations:** 1 Unit Applied Mathematics and Computer Science (MIA Paris-Saclay), INRAE AgroParisTech Université Paris-Saclay, 91120 Palaiseau, France; 2 European Commission, Joint Research Centre (JRC), Ispra, Italy; 3 Department of Soil, Plant and Food Sciences (DiSSPA – Entomology and Zoology), University of Bari, 70126 Bari, Italy

**Keywords:** Agricultural policy, Bias, False discovery, Fast-track synthesis, Meta-analysis, Vote counting

## Abstract

Statistical synthesis of data sets (meta-analysis, MA) has become a popular approach for providing scientific evidence to inform environmental and agricultural policy. As the number of published MAs is increasing exponentially, multiple MAs are now often available on a specific topic, delivering sometimes conflicting conclusions. To synthesise several MAs, a first approach is to extract the primary data of all the MAs and make a new MA of all data. However, this approach is not always compatible with the short period of time available to respond to a specific policy request. An alternative, and faster, approach is to synthesise the results of the MAs directly, without going back to the primary data. However, the reliability of this approach is not well known. In this paper, we evaluate three fast-track methods for synthesising the results of MAs without using the primary data. The performances of these methods are then compared to a global MA of primary data. Results show that two of the methods tested can yield similar conclusions when compared to global MA of primary data, especially when the level of redundancy between MAs is low. We show that the use of biased MAs can reduce the reliability of the conclusions derived from these methods.

**Supplementary Information:**

The online version contains supplementary material available at 10.1186/s13750-023-00309-y.

## Background

Systematic review and meta-analysis (MA) are essential tools for the synthesis of knowledge in many fields, particularly in medical sciences but also in ecology and environmental sciences [12]. A systematic review involves the exhaustive assembly, evaluation and synthesis of most relevant studies dealing with a specific question. It should be based on a detailed protocol limiting the bias and favoring a transparent and reproducible approach [5]. Systematic review including meta-analysis aims to provide quantitative information from a set of relevant primary studies. The main output of most MA is a mean effect size measuring the effect of an intervention on an outcome of interest relatively to a comparator. Together with its confidence interval, the mean effect size indicates whether the intervention has a significantly positive or negative effect on the outcome and provide information on the average magnitude of the effect, based on experimental or observational data. Note that, when several interventions and comparators are considered in a given MA, several mean effect sizes are usually estimated and reported.

In environmental and agricultural sciences, it has been recognized that MA has several advantages over the use of single studies [18, 24]: (i) MA increases the statistical power; (ii) MA allows the assessment of the level of generality of local experimental results; (iii) MA helps to analyze the variability of the performances of farming practices across a range of bio-geographical, environmental and farm management conditions; and (iv) MA may shed light on seemingly contradictory research outcomes. Numerous MAs have been published to quantify the impacts of a large range of farming practices and farming systems (e.g., cover crops, intercropping, agroforestry, organic farming and conservation agriculture) on many outcomes related, in particular, to crop production, water and soil quality, biodiversity, pest- and disease-control, and greenhouse gas emissions [3, 17, 24, 29–31].

As the number of published MAs is increasing exponentially [2, 12], multiple MAs are now often available on a specific topic, reporting a broad range of results with sometimes conflicting conclusions. For example, more than 10 MAs (each including several dozen studies) have been conducted to evaluate the impact of agroforestry on soil organic carbon compared to arable systems without trees [14, 15]. It is therefore often necessary to consider the results of multiple MAs in order to answer a given question, on the basis of all available evidence. To synthesise multiple MAs, a first approach is to retrieve the original individual effect sizes (or original experimental data) of all the MAs and make a new MA of the whole dataset. This approach can be quickly implemented only if all individual effect sizes (and their standard errors) used in each first order meta-analysis are available but, in practice, this is often not the case [2, 26]. When these data are not available, they need to be extracted from the individual studies and this approach then becomes time consuming and not always compatible with the time available to respond to a specific policy advice request. Lack of time is often seen as a major barrier to the use of scientific evidence by policy makers [7, 23]. Often the demands of policy makers have to be met within a few days or a few weeks, which does not leave enough time to extract the primary data taken into account by the MAs. This is particularly the case for requests from the European Commission (e.g., DG Agri) concerning the environmental impact of agricultural practices (European Commission, [10]). Indeed, in order to identify effective sustainable techniques and justify public subsidies supporting specific farming practices, it is necessary to provide the decision-makers with robust scientific evidence in a short period of time. We are considering here a real situation where decision-makers demand a response within a few weeks and where several MAs of good quality have already been published (Makowski et al. [19]).

To provide a rapid approach of evaluating interventions (e.g., agricultural practices, nature restoration techniques) based on a large number of experiments, an alternative approach is to synthesize the results of several MAs without going back to the original primary data. This is for example the case in vote-counting of MA results or in second-order MAs, which has gained in popularity, especially in agricultural sciences and ecology [3, 6, 11, 31]. These approaches present several practical advantages, in particular for the stakeholders involved in policy decision-making. However, several potential limitations have been identified for some of these fast-track methods (e.g., partial redundancy between MAs, lack of statistical power, risk of bias) and the reliability of their results is barely studied. Thus, there is a need to assess the performances of different time-saving methods for synthesizing results of MAs in order to inform effective environmental policies.

The evaluation of these methods is all the more important since syntheses of MAs are potentially subject to different types of bias. Although the systematic review methodology was designed to both reduce bias in syntheses and assess risk of bias in primary datasets, different types of bias may occur at different stages of the process, i.e., in the individual experiments included in a systematic review (e.g., bias arising from the randomisation process, bias in measurement of the outcome), in the individual meta-analyses (in particular, publication bias resulting from the selection of studies during the publication process), and during the synthesis of the MAs (e.g., selection of MAs with special characteristics). These biases can be cumulative. Indeed, since the conclusions drawn in a meta-analysis depend on the results of the included studies, if the results of the individual studies are biased, a meta-analysis of these studies could produce a misleading conclusion. Subsequently, the syntheses of biased MAs could lead to an overestimation or underestimation of the magnitude of the effect. Several tools have been developed to detect the existence of bias [27, 34] and even to correct biased meta-analyses [9, 22]. However, as it is not possible to totally eliminate bias, it is still important to assess the impact of these biases on the performance of MA synthesis methods. Among the different types of bias, publication bias has attracted a special attention because it can lead to a strong under-estimation or over-estimation of the true mean effect sizes [35].

The objective of this paper is to contribute to the field of evidence synthesis by comparing the performances of three fast-track methods for synthesising the results of MAs without using the original primary data, namely (i) second-order MA (MA of mean effect sizes of first-order MAs) (SOMA), (ii) single most accurate first-order MA (MA reporting the mean effect size with the lowest coefficient of variation) (MAMA), (iii) majority of first-order MAs results (vote counting of MAs results reporting positive, negative, and non-significant effect) (COMA). These methods are reflective of fast-track methods commonly used in practice [3, 6, 8, 11, 31]. Using simulated data [20], we compare the results of these three methods to the results obtained by a global MA of primary data (REMA, Cooper and Koenda, [8]). It should be noted that we focus here on MAs evaluating the effect of an intervention versus a comparator, as this type of MAs is widespread and is often used by decision-makers to assess the performance of a given intervention.

Our results show that the method SOMA performs well detecting an existing effect, but leads to a relatively high rate of false discovery (risk of wrongly concluding that an effect exists) in case of high redundancy of primary data between first-order MAs (i.e., when several MAs have in common a high proportion of studies). The method MAMA leads to biased estimates even in the absence of publication bias, due to its tendency to select extreme mean effect sizes. Finally, when the sample size of each MA is small, the method COMA tends to miss existing effects due to a lack of statistical power, but it has a very low false discovery rate and can thus be trusted when concluding to a positive or negative effect. Our results also show that the existence of publication bias can reduce the reliability of the conclusions of these methods under certain conditions. Overall, this study shows that second-order MA and majority-results can yield similar conclusions when compared to global MA of primary data, especially when the level of redundancy between first-order MAs is low. However, when practically possible, global MA of the original primary studies (REMA) should remain the preferred method as it reduces the risk of erroneous conclusions.

## Methods

### Methods considered for synthesizing the results of meta-analyses

We consider three fast-track methods and compare them to a reference method used as a benchmark (Table 1). The first method (SOMA) consists in conducting a second-order MA based on the mean effect sizes produced by a series of first-order MA. Instead of analysing the primary data, the method SOMA computes a weighted average of the mean effect sizes produced by the first-order MAs. Thus, if the results of *K* first-order MAs are available, SOMA summarises the *K* corresponding estimated mean effect sizes by computing the average of these *K* values, using their respective variances as weights. The result of SOMA is a new single overall mean effect size summarising the whole set of *K* first-order MA. Formally, let define the *K* first-order estimated mean effect sizes as ${\Delta }_{1},{\Delta }_{2},{\dots , \Delta }_{k}\dots ,{\Delta }_{K}$ provided by the *K* MAs, and their standard errors as${\sigma }_{1},{\sigma }_{2},{\dots , \sigma }_{k}\dots ,{\sigma }_{K}$. Assuming a Gaussian distribution and independence between the ${\Delta }_{k}$, *k* = 1,…,*K*, and following the standard procedure commonly used for MA (Borenstein et al. [4]), SOMA estimates an overall mean effect size as ${\Delta }_{SOMA}=\frac{\sum_{k}^{K}{ {w}_{k}\Delta }_{k}}{\sum_{k}^{K}{w}_{k}}$, where the weight ${w}_{k}$ is defined as ${w}_{k}=\frac{1}{{{\tau }^{2}+\sigma }_{k}^{2}}$, with ${\tau }^{2}$ the variance measuring the heterogeneity between the first-order MAs*.* SOMA also computes the lower and upper bounds of the 95% confidence interval of ${\Delta }_{SOMA}$ as ${{L}_{SOMA}=\Delta }_{SOMA}-1.96{\delta }_{SOMA}$ and${{U}_{SOMA}=\Delta }_{SOMA}+1.96{\delta }_{SOMA}$, where ${\delta }_{SOMA}$ is a standard error of ${\Delta }_{SOMA}$ computed as the inverse of the square root of the sum of the weight ${w}_{k}$, as in standard MA (Borenstein et al. [4]). Based on SOMA, the effect is then be considered as significantly positive if ${L}_{SOMA}>0$, significantly negative if ${U}_{SOMA}<0$, and not significant if ${L}_{SOMA}<0<{U}_{SOMA}$. This method is attractive as it allows one to summarize the results of the *K* first-order MAs by a single mean value (${\Delta }_{SOMA}$) and to describe the uncertainty by a single confidence interval [${L}_{SOMA}$, ${U}_{SOMA}$]. However, the hypothesis of independence of the ${\Delta }_{k}$, *k* = 1,…,*K* is violated if the first-order MAs were performed from overlapping datasets (i.e., datasets sharing some primary studies).

**Table 1:** Methods considered to synthesize results of meta-analyses

Name	Inputs	Use data from primary studies	Procedure	Outcome
Second-order meta-analysis (SOMA)	Mean effect size of 1st order MAs and their standard errors	No	Weighted average of the means effect sizes reported by the MAs	Overall mean effect size and its confidence interval
Most accurate meta-analysis (MAMA)	Mean effect size of 1st order MAs and their standard errors	No	Select the most accurate MA based on coefficient of variation	Mean effect size and confidence interval of the most accurate 1^st^ order MA
Counting of meta-analysis results (COMA)	Confidence intervals of mean effect sizes of 1st order MAs	No	Count the number of significant positive and negative effects, and of non-significant effects	Qualitative score (Positive effect, Negative effect, No effect)
Reference meta-analysis (REMA)	Individual effect sizes of primary studies and their standard errors (or original data)	Yes	Global MA of all individual studies	Overall mean effect size and its confidence interval

The second method (MAMA) consists in selecting a single MA among the *K* first-order MAs. Here, we select the MA leading to the most accurate mean effect size estimate, where the accuracy is measured through a coefficient of variation (CV) defined as the ratio of the standard error of the estimated mean effect size to the absolute value of the estimated mean effect size, i.e., ${CV}_{k}=\frac{{\sigma }_{k}}{\left|{\Delta }_{k}\right|}$, *k* = 1, …, *K*. The coefficient of variation is a standard measure of accuracy (the lower, the most accurate). In the context of MA, CV expresses the accuracy of the estimated mean effect size as a single number, resulting from the combination of several factors such as the number of individual studies, the accuracy of each of these studies (depending itself on the number of data and their dispersion), the heterogeneity among studies, and the size of the effect. With MAMA, the whole set of first-order MA is summarized by the mean effect size (and its 95% confidence interval) reported in the MA with the lowest CV, further noted as ${\Delta }_{MAMA}$, ${{L}_{MAMA}}$ and ${U}_{MAMA}$. Based on MAMA, the effect is considered as significantly positive if ${L}_{MAMA}>0$, significantly negative if ${U}_{MAMA}<0$, and not significant if ${L}_{MAMA}<0<{U}_{MAMA}$

The third method (COMA) is based on a vote counting procedure. Instead of combining the *K* first-order mean effect sizes into an overall mean effect size as in SOMA, the method COMA allocates the *K* first-order MAs in three categories according to the 95% confidence intervals of the estimated first-order mean effect sizes. Let note the lower and upper bounds of confidence interval associated with the mean effect size reported by the k^*th*^ MA as ${{L}_{k}=\Delta }_{k}-1.96{\sigma }_{k}$ and ${{U}_{k}=\Delta }_{k}+1.96{\sigma }_{k}$, respectively. The k^*th*^ meta-analysis is allocated to the positive category if ${L}_{k}>0$, to the negative category if ${U}_{k}<0$, and to the no effect category if ${L}_{k}<0<{U}_{k}$. The number of MAs falling in each category is then counted and the category with most votes is identified. This approach does not quantify any effect size but allows one to categorize the effect among three categories, namely positive (majority of significantly positive first-order mean effect size), negative (majority of significantly negative), or no effect (majority of no effect).

Finally, the reference method (REMA) consists in performing a meta-analysis of all the individual studies taken into account by the *K* first-order MAs. Thus, if each MA is based in *N* studies and if all studies are different, REMA estimates a mean effect size based on the $K\times N$ primary studies. If some of the studies are common among the* K* MAs, the redundant primary studies are removed before the analysis and the total number of primary studies used by REMA is then lower than $K\times N$. As with SOMA, the result of REMA is an overall mean effect size and its corresponding 95% confidence interval ${\Delta }_{REMA}$, ${{L}_{REMA}}$ and ${U}_{REMA}$. Based on REMA, the effect is then be considered as significantly positive if ${L}_{REMA}>0$, significantly negative if ${U}_{REMA}<0$, and not significant if ${L}_{REMA}<0<{U}_{REMA}$. Note that, contrary to the other methods, REMA relies on the primary data, while SOMA, MAMA, and COMA do not request this type of data and rely only on the results provided by the MAs. Compared to SOMA, REMA has the advantage to avoid the use of redundant studies (i.e., primary studies shared by several first-order MAs). However, this approach requires the extraction of all data published in primary studies and thus requires more working time than SOMA.

### Simulations assuming that the MAs are unbiased

Simulated data [20] are traditionally used to compare the performance of different statistical methods (e.g., methods used to estimate some parameters of interest, for example a mean effect size). Simulated data are generated with an explicit statistical model and ‘true’ parameter values chosen by the scientists conducting the assessment. The statistical methods considered are applied to the simulated data and their results are compared to the true values. This approach is a standard practice in statistics because it offers a practical way to compare estimated parameter values to true parameter values. Such a comparison is impossible with real data because the true parameter values are unknown in real case studies.

In order to explore a larger diversity of scenarios, datasets were simulated using a hierarchical statistical model widely used in MA [4, 13], defined as ${y}_{i}={\theta }_{i}+{\varepsilon }_{i}$, with ${\theta }_{i}\sim N\left(\mu ,{\sigma }_{\theta }^{2}\right)$ and ${\varepsilon }_{i}\sim N\left(\mu ,{\sigma }_{\varepsilon i}^{2}\right)$. In this model, ${y}_{i}$ is the individual effect size (typically, a log ratio) reported in the ith study. The parameter $\mu$ is the true mean effect, i.e., the quantity that we want to estimate as accurately as possible or, at least, classify in three categories “positive” ($\mu >0$), “negative” ($\mu <0$), “no effect” ($\mu =0$). The variance ${\sigma }_{\theta }^{2}$ represents the between-study variance of the true effect size of the i*th* study (${\theta }_{i})$, and ${\sigma }_{\varepsilon i}^{2}$ is the within-study variance (each study is assumed to have a specific variance in order to reflect the fact that some studies may be more accurate than others).

**Fig. 1:**
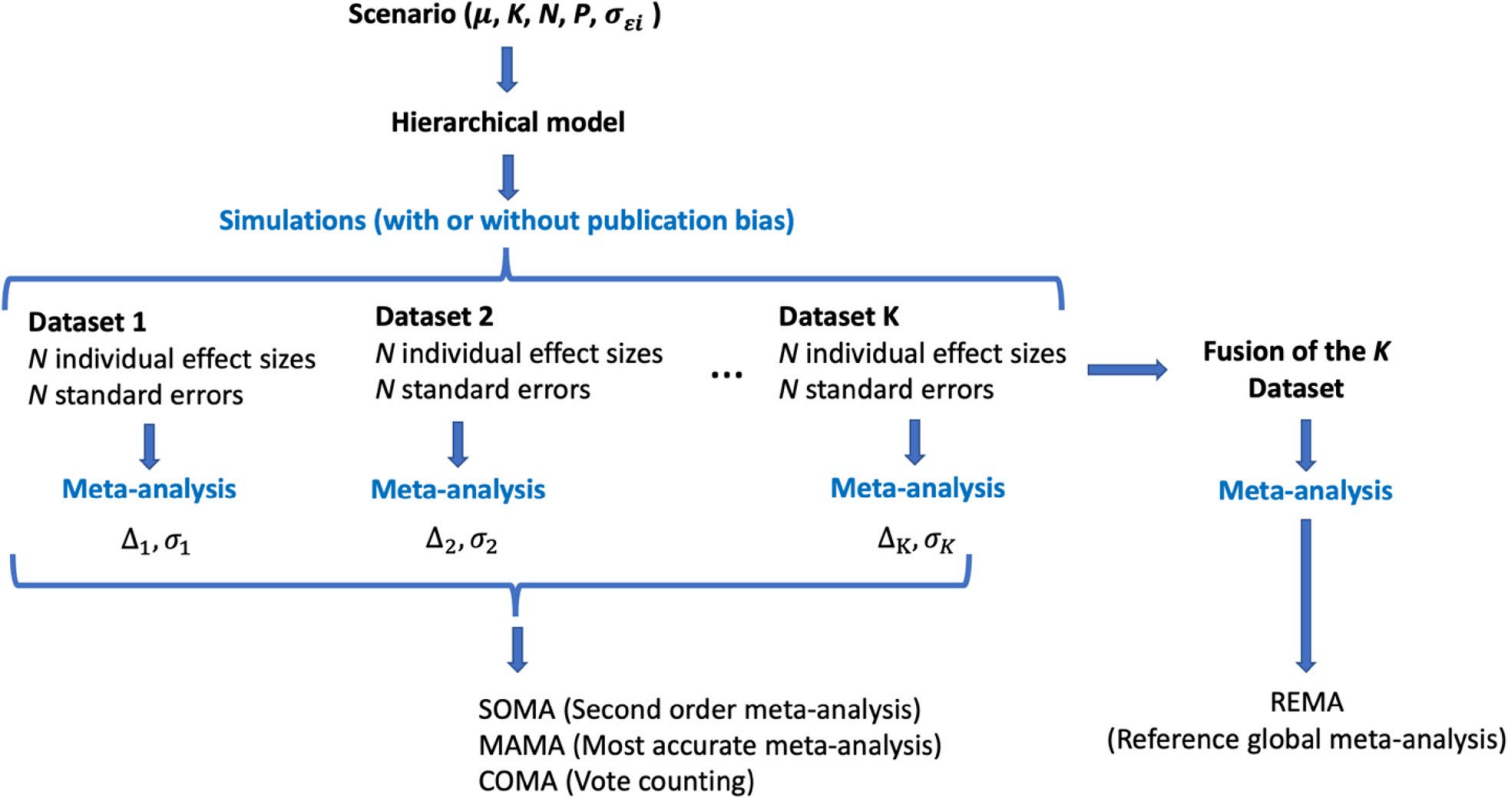
Implementation of the methods SOMA, MAMA, COMA, and REMA (see Table 1) to simulated data. For a given scenario (characterized by a true mean effect size $\mu$, a number of datasets (*K*), a number of data per dataset (*N*), a level of redundancy (*P*), and a level of precision), a hierarchical Gaussian model is used to generate *K* datasets, each including *N* data (effect sizes and standard errors). A 1st order MA is performed using each dataset in turn, generating* K* mean effect sizes (${\Delta }_{1},{\Delta }_{2},{\dots , \Delta }_{k}\dots ,{\Delta }_{K}$) and standard errors $({\sigma }_{1},{\sigma }_{2},{\dots , \sigma }_{k}\dots ,{\sigma }_{K}$). These quantities are used to implement the methods SOMA, MAMA, and COMA (see text). In addition, the *K* datasets are merged to produce a single global dataset used to implement the method REMA

The statistical model is used to generate datasets and compare the methods SOMA, MAMA, COMA and REMA, as described in Fig. 1. The datasets are generated according to different scenarios, each characterized by a true mean effect size ($\mu =-0.69, -0.29, 0, 0.22, 0.41$, expressed as a log ratio, corresponding to relative change of − 50%, − 25%, 0%, + 25%, + 50%, respectively), a number of first-order MAs (*K* = 3, 5, 10), a number of primary data in each first-order MA (*N* = 10, 15, 25, 50), a proportion of common data among the *K* first-order MAs (*P* = 0, 10, 25, 50%), and a level of precision of primary data (low, medium, high within-study variances ${\sigma }_{\varepsilon i}^{2}$). The total number of scenarios is thus 5 × 3 × 4 × 4 × 3 = 720.

At each iteration for a given scenario, *K* virtual datasets are generated using the statistical model defined above. Each one of these *K* datasets is specified such as it includes *N* data (*N* pairs of ${y}_{i}$ and ${\sigma }_{\varepsilon i}^{2}$), with a proportion *P* of common data among the *K* datasets. A MA is then performed using each dataset in turn with a random-effect model, leading to *K* estimated mean effect sizes (${\Delta }_{1},{\Delta }_{2},{\dots , \Delta }_{k}\dots ,{\Delta }_{K}$) and their standard errors $({\sigma }_{1},{\sigma }_{2},{\dots , \sigma }_{k}\dots ,{\sigma }_{K}$) (Fig. 1). These results are then used to implement the method SOMA, MAMA, and COMA as explained above. Finally, the *K* subsets of data are merged together and used to implement a single global meta-analysis—after the removal of the *P*% of redundant data—in order to implement the method REMA (Fig. 1). The procedure is repeated 100 times for each scenario. The computations were done with the R software (R Core Team, 2021). The code used to simulate data is shown in Additional file 1: A and all codes used in the analysis are available at https://github.com/davemakowski/CodePaper2ndOrderMAs.

### Simulations of biased first-order MAs

Publication bias may arise from the preferential publication of statistically significant studies and/or of studies with results in a particular direction (positive or negative). In order to better understand the impact of publication bias on the results of the methods presented in Table 1, we made additional simulations considering three types of publication bias, successively: (i) bias resulting from the selective publication of studies with statistically significant individual effects (negative or positive), (ii) bias resulting from the selective publication of studies with statistically significant negative effects, (iii) bias resulting from the selective publication of studies with statistically significant positive effects. With the first type of bias, we consider that an individual study showing non-significant effect is not published, while an individual study showing a significant effect size is published, whatever the direction of the effect. With the second (third) types of bias, we consider that an individual study is published only if it shows a significant negative (positive) effect. The conditions of publication are thus more restrictive with the second and third types of bias than with the first one. 

The datasets are generated considering 27 scenarios, each characterized by a true mean effect size ($\mu =-0.69, -0.29, 0$, expressed as a log ratio, corresponding to relative change of − 50%, − 25%, and 0%, respectively), a number of first-order MAs (*K* = 3, 5, 10), and a type of publication bias (i, ii, or iii, as explained above). The number of primary data in each first-order MA was set to *N* = 50, *P* was set equal to 0, and the level of precision of primary data was set to medium within-study variances in all scenarios. The same procedure as above is implemented to generate *K* datasets each including *N* data (pairs of ${y}_{i}$ and ${\sigma }_{\varepsilon i}^{2}$), at each iteration. However, here, the data are generated to reflect publication bias, considering a statistical significance at a level of 5%. For publication bias 1, the data are generated such as ${y}_{i}$ + 1.96 ${\sigma }_{\varepsilon i}^{2}$ < 0 or ${y}_{i}$—1.96 ${\sigma }_{\varepsilon i}^{2}$ > 0 (i.e., significantly positive or negative effect). For publication bias 2 (3), the data are generated such as ${y}_{i}$ + 1.96 ${\sigma }_{\varepsilon i}$ < 0 (${y}_{i}$—1.96 ${\sigma }_{\varepsilon i}$ > 0). Note that, here, the third type of bias is the most extreme because it implies that the studies are deliberately selected to show results that are opposite to the truth. The procedure is repeated 100 times for each scenario. We did not perform simulations for positive values of $\mu$ because the results would have been symmetrical and the conclusions unchanged. 

### Method comparison

SOMA, MAMA, COMA and REMA are compared using four criteria, namely the probability of correct conclusion (PCC), the bias of the estimated mean effect size (BES), the root mean square error of the estimated mean effect size (RMSE), and the coverage of the confidence interval of the estimated mean effect size (CCI). These criteria are computed for each scenario, as explained below.

For each scenario, we obtain a series of 100 estimated mean effect size estimates and confidence intervals for the SOMA, MAMA, and REMA methods. The 100 confidence intervals of SOMA, MAMA and REMA are used to allocate the 100 corresponding estimated mean effect sizes to categories selected among positive, negative, and no effect. For COMA, we obtain the majority results of the *K* MAs, as explained above. A good classifier would correspond to a classifier selecting the true category (“negative”, “positive” or “no effect”, depending on the scenario considered) as often as possible. Consequently, the relevance of the categories generated by each method is evaluated by calculating PCC as the proportion of the 100 categories corresponding to the true category. The probability of correct conclusion PCC assesses the ability of the methods to determine the true direction of the effect of the tested intervention relatively to the comparator, but it does not evaluate the accuracy of the estimated mean effect sizes obtained with SOMA, MAMA, and REMA. In order to do so, we calculate three other criteria; the bias BES defined as the difference between the ‘true’ mean effect size (*μ*) and the average of the 100 estimated mean effect sizes obtained with SOMA, MAMA and REMA, the RMSE defined as the root square of mean of the squared difference between the 100 estimated mean effect size and the value of *μ*, and the coverage of the 95% confidence interval CCI defined as the proportions of the 100 confidence intervals including *μ*. Note that bias and RMSE values are related to each other because the bias is one of the components of the RMSE (Mean squared error = Bias^2^ + Variance). Thus, an increase (decrease) of absolute bias tends to increase (decrease) the RMSE.

The criteria PCC, BES, RMSE, and CCI are computed considering each of the scenarios defined above in turn. The results obtained assuming no publication bias are first presented, and are then compared with the results obtained with publication bias. Other types of bias are not considered.

## Results

### Results obtained when the first-order MAs are unbiased

**Fig. 2:**
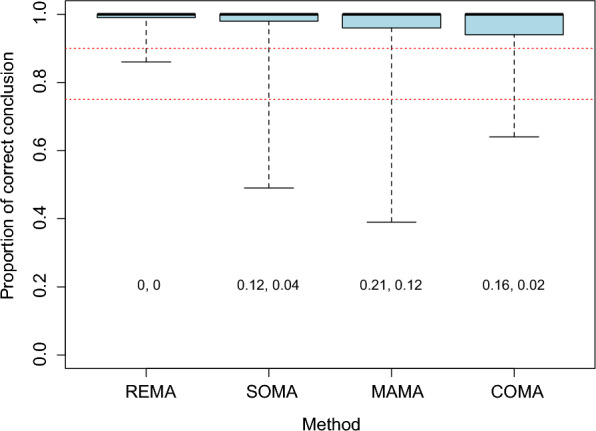
Proportion of correct conclusion obtained with the four methods. Each boxplot describes the distributions across 720 scenarios characterized by different numbers of first-order MAs, data per MA, and different levels of redundancy between first-order MAs (see Methods). Red dashed lines indicate the proportions 0.75 and 0.9. The numbers displayed below the boxplots indicate the proportion of scenarios where the proportion of correct conclusion is lower than 90% and 75%, respectively. The proportion of correct conclusion PCC assesses the ability of the methods to determine the true direction of the effect of the tested intervention relatively to the comparator

**Fig. 3:**
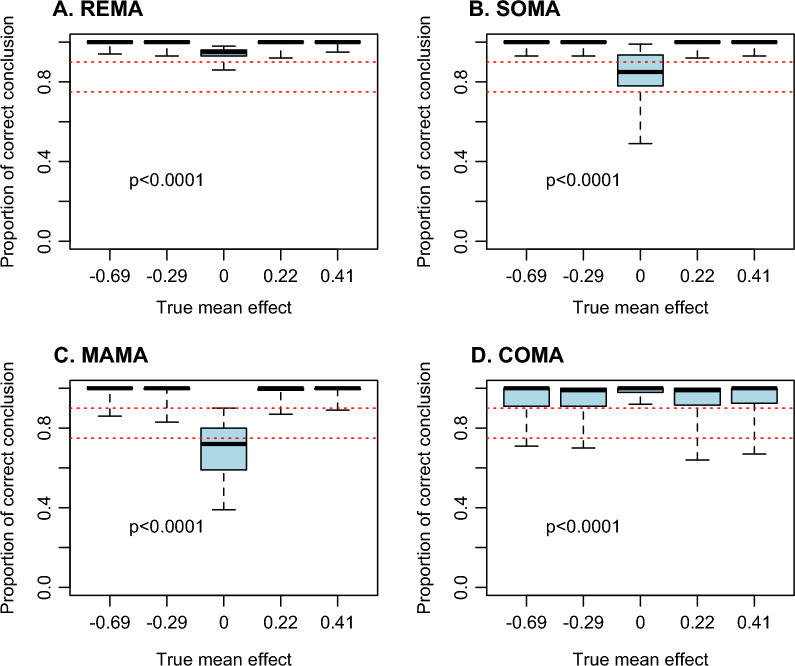
Proportion of correct conclusion obtained with the four methods as the function of the true mean effect sizes. Each boxplot describes the distribution of results across different scenarios. Red dashed lines indicate the proportions 0.75 and 0.9. Values of *p* indicate the significance of the relationship between the proportion of correct conclusion and the true mean effect. The proportion of correct conclusion PCC assesses the ability of the methods to determine the true direction of the effect of the tested intervention relatively to the comparator

Figure 2 shows that all methods lead to high proportions of correct conclusions (PCC) in most scenarios, but with some differences between methods. The PCC values of all methods are above 0.9 in more than 75% of the scenarios, but the PCC values of REMA are less variable than those of the other methods, in particular compared to COMA and MAMA. Thus, while PCC is lower than 0.9 in 0% and 12% of the scenarios with REMA and SOMA respectively, these percentages reach 16% and 21% with COMA and MAMA respectively (Fig. 2). Interestingly, values of PCC are highly dependent on the true value of the mean effect size (Fig. 3). With REMA, SOMA and MAMA, the proportion of correct conclusions is thus very close to one when the true mean effect size is different from zero (i.e., when there is a true positive or negative effect) but this proportion is lower when the true mean effect is zero (i.e., true no effect). In case of absence of effect, the risk of false discovery (concluding that there is an effect while there is no effect in reality) can reach high values in a relatively large proportion of scenarios with SOMA and MAMA, and more particularly with MAMA for which PCC is lower than 0.75 (i.e., more than 25% chance of false discovery) in more than 50% of the scenarios tested (Fig. 3C). The results obtained with COMA are opposite. Indeed, with this method, values of PCC are very close to one when the true mean effect is zero but tend to be substantially lower than one in case of true positive or negative mean effects (Fig. 3D). These results indicate that the conclusions of COMA are more reliable in case of absence of effect (almost no risk of false discovery with COMA in case of true absence of effect) but that, on the contrary, the conclusions of REMA, SOMA and MAMA are more reliable in case of true positive or negative effects. The values of PCC decrease significantly with the proportion of common data, but the magnitude of the decline is small, as shown by the median PCC, which remains well above 0.9 with all the proportions of common data considered (even 50%) and all methods (Additional file 1: B).

**Fig. 4:**
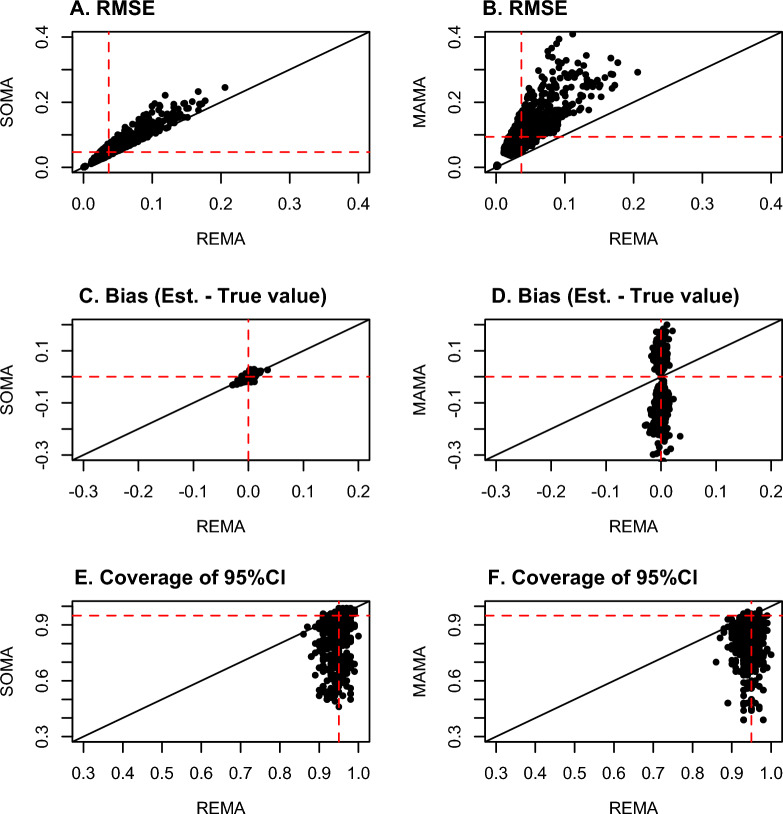
Evaluation of the accuracy of the mean effect sizes estimated using the methods SOMA and MAMA as compared to REMA, according to the Root Mean Square Error (RMSE), Bias (Estimated value—True value), and coverage of the 95% confidence interval (CI). Each point corresponds to one scenario. Red dashed lines indicate the median value of RMSE (**A**, **B**), zero bias (**C**, **D**), the target value 0.95 of the confidence interval (**E**, **F**)

**Fig. 5:**
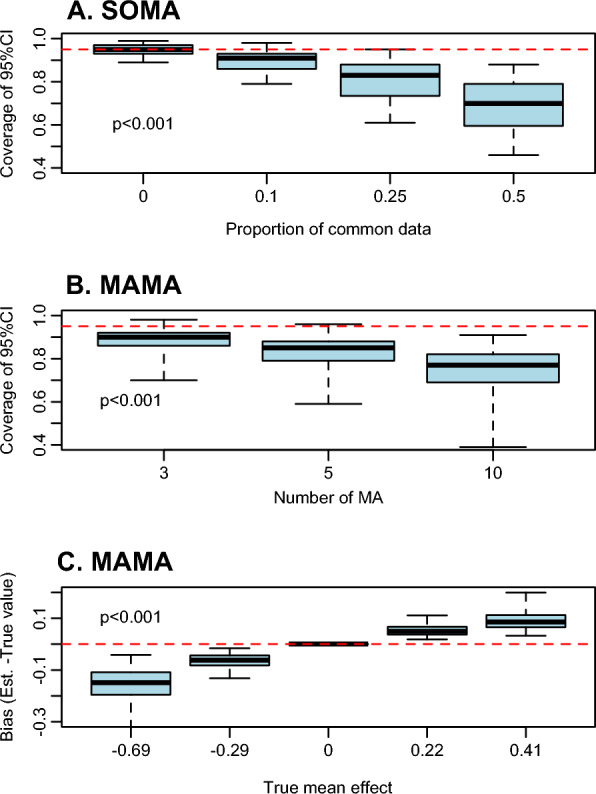
Factors explaining the coverage of confidence intervals obtained with the methods SOMA (**A**) and MAMA (**B**), and explaining the bias of MAMA (**C**). The red dashed lines indicate the target optimal value (0.95) of the confidence interval (**A**, **B**), and zero bias (**C**). The *p* values indicate the significance of the relationships. Results obtained with simulated data

Figures 4–5 show the results of the assessment of the accuracy of the quantitative mean effect size estimated with REMA, SOMA and MAMA. The RMSE values of REMA and SOMA are strongly correlated, but the RMSE of SOMA tends to be slightly higher than the RMSE of REMA (Fig. 4A). Both REMA and SOMA exhibit near-zero bias (Fig. 4C). The RMSE values of MAMA are on average twice as high as REMA values (Fig. 4B). Moreover, the bias of MAMA can be very either highly positive or negative (Fig. 4D), revealing that the mean effect sizes estimated with this method are often higher or smaller than the true values. Whether the bias of MAMA is positive, negative or close to zero depends on the true mean effect size (Fig. 4C). While the bias is zero in case of true absence of effect, the method MAMA tends to overestimate (underestimate) the effect size in case of true positive (negative) effect (Fig. 4C). In other words, the mean effect sizes estimated by MAMA tend to be too extreme (either positively or negatively). Note that, in Figs. 4–5, publication bias and bias of individual studies are assumed to be equal to zero. The only type of bias considered here is that induced by the statistical procedures.

The coverage levels of the confidence intervals obtained with REMA are close to 0.95. On the contrary, the coverage levels obtained with SOMA and MAMA are often lower than this value. This result reveals that the confidence intervals obtained with SOMA and MAMA tend to be too narrow (Fig. 4E, F, Fig. 5). The coverage levels of SOMA are significantly impacted by the scenario characteristics, especially by the proportion of common data among first-order MAs (see Additional file 1: C and Fig. 5A). With SOMA, the coverage levels are very close to zero in case of absence of common data, while they become much lower than 0.95 when the proportion of common data is high (Fig. 5A). With MAMA, the coverage levels are significantly related to the number of first-order MAs, and tend to become too low when the number of MAs is high (Fig. 5B).

### Impact of publication bias

**Fig. 6:**
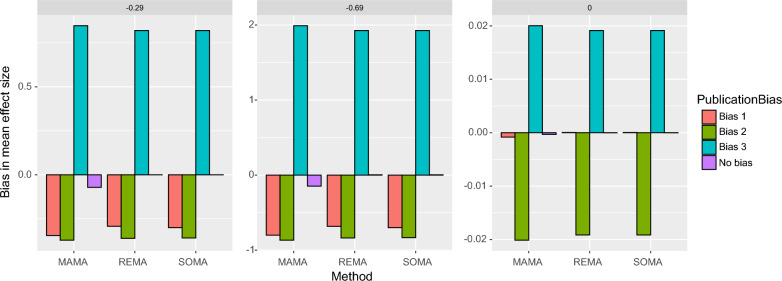
Bias in mean effect sizes estimated by MAMA, REMA, and SOMA (Estimated—True value), with and without publication bias. Three types of publication bias are considered, bias 1 (only individual studies showing significant effects are published), bias 2 (only individual studies with significantly negative effects are published), and bias 3 (only individual studies with significantly positive effects are published). The computations were performed assuming a true mean effect size equal to − 0.29 (loss of − 25%, left), − 0.69 (loss of − 50%, middle), or zero (no effect, right)

Figure 6 shows the bias of mean effect sizes estimated with the methods MAMA, REMA, and SOMA, with and without publication bias in first-order MAs. Clearly, the presence of a publication bias has an impact on the mean effect sizes estimated by the three synthesis methods, but the level of impact depends on the method, on the type of publication bias, and on the true effect. COMA is not considered here because this method does not provide quantitative mean effect size estimate.

When the true effect is negative (− 0.29 or − 0.69), the publication bias of types 1 (“only individual studies showing significant effects are published”) and 2 (“only individual studies with significant negative effects are published”) both induce a negative bias in the mean effect sizes estimated with MAMA, REMA, and SOMA. It means that, with publication bias 1 and 2, the synthesis of first-order MAs tends to produce mean effect sizes that are in the right direction (i.e., negative effects, here) but too extreme (i.e., too strongly negative). On the contrary, publication bias of type 3 (“only individual studies with significant positive effects are published”) induces a positive bias in mean effect sizes for all methods, i.e., mean effect sizes tend to be in the opposite direction than the true value (i.e., positive effect instead of negative). The reason is that, with publication bias 1 and 2, the individual effect sizes selected for the first-order MAs are more extreme than those available in the absence of publication bias, but remain in the right direction in most cases (see Additional file 1: D for an example of a simulated sample of individual effect sizes). The impact of publication bias 2 is stronger than the impact of publication bias 1 because, while both significantly positive and negative individual effect sizes are selected with publication bias 1, only significantly negative individual effect sizes are selected with publication bias 2. With publication bias 3, it is assumed that only significantly positive individual effects are selected, resulting in positive estimated first-order mean effect sizes instead of negative (see Additional file 1: D for an example of sample of simulated individual effect sizes). This type of publication bias induces a positive bias in the mean effect sizes estimated with MAMA, REMA, and SOMA (Fig. 6).

Interestingly, in absence of publication bias (purple color in Fig. 6), MAMA still shows a small bias, contrary to SOMA and REMA. This is consistent with the results presented in Fig. 4D. This bias is due to the fact that MAMA tends to select extreme first-order MAs, and this type of bias occurs even in the absence of publication bias. However, the level of this bias is lower than the bias obtained in case of presence of publication bias.

When the true effect is zero, publication bias type 1 does not induce any substantial bias on the mean effect sizes estimated with the methods MAMA, REMA, and SOMA (Fig. 6). This is because the individual effect sizes selected under publication bias 1 tend to be equally positive or negative, and thus tend to compensate each other, leading to a first-order mean effect size close to zero. Thus, in case of absence of effect, publication bias 1 does not impact the results of MAMA, REMA, and SOMA substantially. On the contrary, when the true effect is zero, the publication bias 2 and 3 lead to an underestimation and overestimation of the mean effect size, respectively, thus generating biased mean effect sizes (Fig. 6).

**Fig. 7:**
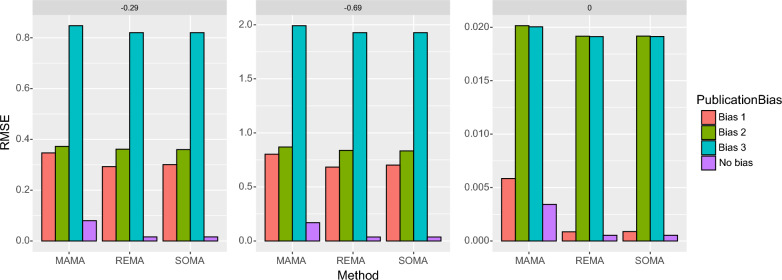
RMSE in mean effect sizes estimated by MAMA, REMA, and SOMA, with and without publication bias. Three types of publication bias are considered, bias 1 (only individual studies showing significant effects are published), bias 2 (only individual studies with significantly negative effects are published), and bias 3 (only individual studies with significantly positive effects are published). The computations were performed assuming a true mean effect size equal to − 0.29 (loss of − 25%, left), − 0.69 (loss of − 50%, middle), or zero (no effect, right)

Figure 7 shows the RMSE obtained with MAMA, REMA, and SOMA with and without publication bias. The RMSE is higher with publication bias than without, but its level depends on the type of publication bias considered. The highest RMSE values are obtained with publication bias 2 and 3, due to the fact that more extreme individual effect sizes tend to be selected in these cases. The RMSE values obtained with publication bias 1 are lower but still higher than the RMSE values obtained without publication bias.

**Fig. 8:**
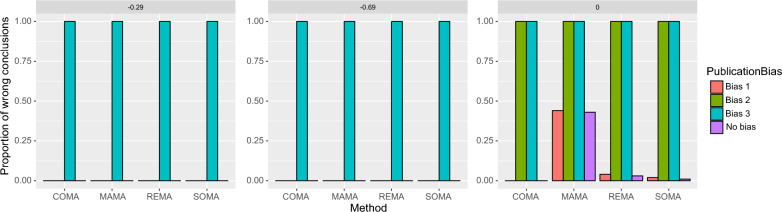
Proportion of wrong conclusions obtained with COMA, MAMA, REMA, and SOMA, with and without publication bias. Three types of publication bias are considered, bias 1 (only individual studies showing significant effects are published), bias 2 (only individual studies with significantly negative effects are published), and bias 3 (only individual studies with significantly positive effects are published). The computations were performed assuming a true mean effect size equal to − 0.29 (loss of − 25%, left), − 0.69 (loss of − 50%, middle), or zero (no effect, right)

Figure 8 shows the proportion of wrong conclusions (i.e., 1-PCC) obtained with COMA, MAMA, REMA, and SOMA, with and without publication bias. The results are contrasted, depending on the true value of the mean effect size and on the type of publication bias, especially on whether individual studies are deliberately selected to show results that are opposite to the truth. When the true value is negative, the proportion of wrong conclusion is zero with publication bias 1 and 2. This is logical because, as mentioned above, publication bias 1 and 2 lead to too extreme estimated values of mean effect sizes but the estimated values still remain in the right direction. In other words, the mean effect sizes estimated with publication bias 1 and 2 indicate significant negative effects and, although the estimated values are too extreme, they lead always to a correct conclusion (a symmetrical result would have been obtained if the true effect was chosen to be positive for data simulation). On the contrary, with publication bias 3, the mean effect sizes provided by the first-order MAs are opposite to the true value and thus systematically lead to the wrong conclusion (i.e., positive effects are estimated while the truth is negative effect). The proportion of wrong conclusion is thus always equal to 1 with publication bias 3.

When the true value is zero, the proportion of wrong conclusion is close to zero with COMA, REMA, and SOMA in case of publication bias of type 1 (Fig. 8). This is because the significant individual effect sizes selected under publication bias 1 tend to be equally positive or negative, and thus compensate each other, leading to a first-order mean effect size generally non-significantly different from zero. With MAMA and publication bias 1, the proportion of wrong conclusion is larger because this method tends to select the most extreme first-order MA available. Consequently, MAMA sometimes selects a first-order MA showing a significantly positive or negative effect only because it has a lower CV, leading to a wrong conclusion. In case of publication bias 2 and 3 and absence of effect (*μ* = 0), the proportion of wrong conclusions is close to 100% with all four methods because the selected individual effect sizes (and resulting first-order MAs) show either significantly negative (publication bias 2) or significantly positive (publication bias 3) results, while no effect exists in the reality. Note that similar results are obtained with the two other values of *K* considered (Additional file 1: E and F).

## Discussion

The number of MAs and systematic reviews published has increased markedly over the past two decades, in particular in medical science [1, 16, 28, 32], in biology (Nagakawa et al. [21]), and more recently in environmental and agricultural science [3]. With the increased number of MAs available, a logical next step is to conduct umbrella reviews of existing MAs in order to synthesize their findings, thereby providing policy makers with robust evidence (Makowski et al. [19]). Until now, very little attention has been paid to methods for synthesizing results from several MAs and it is therefore becoming increasingly important to compare the performance of such methods.

In this study, we have compared four methods (REMA, SOMA, MAMA, COMA) able to determine whether the intervention under consideration has a positive, negative, or no effect relative to its comparator, based on several MAs. Three of these methods (REMA, SOMA, MAMA) allow to quantify the average size of this effect as well, and thus go beyond a qualitative conclusion, while the last one (COMA) only provides a qualitative information about the direction of the effect. The method COMA is thus less informative than the three others as it does not allow to quantify effect sizes, but it is faster to implement as it does not require the extraction of the effect sizes but only whether the MAs show significantly positive, negative, or not statistically significant effect.

Of the three quantitative methods, REMA clearly requires more effort than SOMA and MAMA because it relies on the primary studies and not on the average effect sizes of MAs. To implement REMA, it is therefore necessary to compile data from all primary studies, whereas this is not necessary with SOMA and MAMA. In terms of implementation time, the methods considered here can thus be classified into three categories: the fastest is the COMA method, the SOMA and MAMA methods are intermediate, and the most time consuming is REMA.

The four methods have contrasted performances, both in terms of probability of correct conclusion concerning the existence or non-existence of an effect and in terms of accuracy of the quantitative estimation of the effect size. Their performances also depend on the presence or absence of publication bias, and on the type of publication bias considered.

In case of absence of publication bias, the probability of correct decision is very high with REMA in all situations. Conversely, this probability is lower with both SOMA and MAMA in case of true absence of effect, and also lower with COMA in case of true positive or true negative effect. This means that SOMA and MAMA lead to a higher risk of false discovery (i.e., false conclusion of a "positive" or "negative" effect), while COMA leads to a higher risk of a false "no effect" conclusion. For COMA, the lower probability of a correct conclusion is related to a lack of statistical power. Indeed, with COMA, the results of the first-order MAs are not combined together to obtain an overall, more accurate estimate. The lack of power of COMA is less problematic than the lack of power of the vote counting approach based on individual studies (Borenstein et al. [4], chapter 28) because each first-order MA combines several studies and has thus more power than any single individual studies. Nevertheless, COMA still suffers from a lack of statistical power compared to the three other methods considered here, especially when the sample size of each MA is low. In case of publication bias, the probability of correct decision is generally unchanged with all methods, with two noticeable exceptions; (i) in case of a publication bias leading to the systematic selection of studies showing conclusions opposite to the truth, (ii) in case of absence of effect (true effect equal to zero) and systematic selection of studies showing effects all in the same direction. In all other cases, the probability of correct conclusion is similar with and without publication bias, especially when the publication bias leads to the selection of statistically significant studies without any preference in terms of direction of effect.

The mean effect sizes estimated by three quantitative methods do not have the same level of precision. The best results are obtained with REMA. Performances of SOMA are close, in particular the bias of SOMA and REMA are similar. Results obtained with MAMA are much more biased and the mean effect sizes estimated by this method tend to be too extreme, either too strongly positive or too strongly negative. The poor performance of MAMA is due to the fact that this method is based on the single MA with the lowest coefficient of variation, i.e. on the MA with the lowest ratio of standard error to absolute mean value. For this reason, MAMA tends to select MA with large absolute mean values that can be quite different from the true mean value. Another issue with MAMA and SOMA is that, in some situations, their confidence intervals are too narrow and give an overly optimistic view of the accuracy of the estimated mean effect sizes. With MAMA, the confidence intervals are too narrow when the number of first-order MA is equal to or higher than five. The reason is again related to the fact that MAMA select the single most accurate MA among the set of available MAs. The MA selected with MAMA tends thus to be the first-order MA with most narrow confidence interval among the set of available MAs. Consequently, when the set of first-order MAs is large, the confidence interval of the MA selected by MAMA can be very narrow. With SOMA, the confidence intervals tend to be too narrow when the redundancy between the first-order MAs is high, specifically when the proportion of data in common among the MAs is higher than 25%. In this case, the assumption of independence of the first-order MAs is unrealistic and the confidence intervals computed by the second-order MA is too optimistic. On the other hand, when the redundancy between first-order MAs is low, the coverages of the confidence intervals of SOMA are satisfactory. It is worth noting that the SOMA method also allows for re-estimation of the mean effect sizes of first-order MAs using shrunken estimators (BLUP), as shown by Fox [11]. However, for policy decision support, the overall mean effect across all MAs is more relevant because it summarizes all available information in a single meaningful number.

Finally, it is important to mention that the accuracy of the mean effect sizes provided by MAMA, REMA, and SOMA is impacted by the presence of publication bias. Interestingly, all types of publication bias do not have the same impact. The strongest impact was found with a publication bias selecting studies showing results opposite to the truth, which is probably not a very common type of publication bias. The smallest impact was found with a publication bias selecting studies with statistically significant results, without any preference in terms of direction (either significantly positive or negative). Finally, an intermediate impact level was found with publication bias selecting studies with statistically significant effects in the correct direction. It should be noted that other types of bias may have an impact on the reliability of the results of the methods tested here, in particular biases in the individual experiments included in the first-order MAs.

Based on our results, we can make the following recommendations. In case of low time constraints, REMA is the best option because it leads to the highest probability of correct conclusion and the most accurate quantitative estimates. In case of high time constraints, COMA is an attractive option because it can be used to determine the direction of effect without the need for data extraction. However, COMA does not allow for quantification of effect size and suffers from a lack of statistical power. Finally, in case of medium time constraint and/or when the effect size needs to be quantified, the SOMA method is a relevant choice as it allows to quantify the effect size with low bias and high precision. However, it is important to keep in mind that, in the case of high redundancy between first-order MAs, the confidence intervals computed by SOMA are too optimistic and increase the risk of false discovery. Obviously, the quality of the results of these methods depends on the quality of the first-order MAs. In particular, the use of biased MAs can have an impact on the reliability of the conclusions and it is therefore important to ensure that the selected first-order MAs are of a good level of quality by using explicit quality criteria, such as those proposed by Shea et al. [27] or Beillouin et al. [2].

## Supplementary Information

**Additional file 1.** Code used to generate simulated data and additional results.

## Data Availability

The virtual data were generated using the R code available in the Additional file and in the github repository https://github.com/davemakowski/CodePaper2ndOrderMAs
