# Supplementary material for: Synthesising results of meta-analyses to inform policy: a comparison of fast-track methods
Source: Environ Evid. 2023 Aug 21;12:16. doi: 10.1186/s13750-023-00309-y (PMC11378786; doi:10.1186/s13750-023-00309-y)

Supplementary materials

A. Code (R function) used to simulate virtual data

```
SimulMA2<-function(NumMA,NumS,Prop,MU,K2, Seed) {

library(metafor)
set.seed(Seed)

#Number of simulations
N=100
RESULTS=matrix(NA,nrow=N,ncol=14)

for (k in 1:N) {

#Number of MAs per simulation
#NumMA

#Number of studies per MA
#NumS

#Number of studies in common
#Prop
NumC=NumS*Prop

#True mean effect size
#MU

#Between-study standard deviation
K1=1
Sigma_b=abs(MU)*K1+0.01

#Within-study standard deviation
Sigma_e_min=(abs(MU)*K2+0.01)/2
Sigma_e_max=(abs(MU)*K2+0.01)

#Generation of the studies in common
if (NumC>0) {
Theta_c=rnorm(NumC, MU, Sigma_b)
Sigma_e_c=runif(NumC, Sigma_e_min, Sigma_e_max)
Y_c=rnorm(NumC,Theta_c,Sigma_e_c)
}

#Generation of the other studies
NumS_o=NumMA*(NumS-NumC)
Theta_o=rnorm(NumS_o, MU, Sigma_b)
Sigma_e_o=runif(NumS_o, Sigma_e_min, Sigma_e_max)
Y_o=rnorm(NumS_o,Theta_o,Sigma_e_o)

#Storage of results
MU_est=rep(NA,NumMA)
LB=rep(NA,NumMA)
UB=rep(NA,NumMA)
SE=rep(NA,NumMA)

#1st order MA

for (i in 1:NumMA) {

 DATA_i=data.frame(Y=Y_o[(NumS*(i-1)+1):(NumS*i)],V=Sigma_e_o[(NumS*(i-1)+1):(NumS*i)]^2)
 if (NumC>0) {
  DATA_i=data.frame(Y=c(Y_c,Y_o[((NumS-NumC)*(i-1)+1):((NumS-NumC)*i)]),V=c(Sigma_e_c^2,Sigma_e_o[((NumS-NumC)*(i-
1)+1):((NumS-NumC)*i)]^2))
 }

 Mod_i=rma(Y,V,data=DATA_i)
 MU_est[i]=Mod_i$b
 LB[i]=Mod_i$ci.lb
```

```
  UB[i]=Mod_i$ci.ub
  SE[i]=Mod_i$se
  }

#2nd order MA (SOMA)

DATA2=data.frame(Y2=MU_est,V2=SE^2)
#print(DATA2)
Mod.2=rma(Y2,V2,data=DATA2)
MU2=Mod.2$b
LB2=Mod.2$ci.lb
UB2=Mod.2$ci.ub
SE2=Mod.2$se

#Vote counting of 1st order MA (COMA)
Vote=LB
Vote[LB>0]=1
Vote[UB<0]=(-1)
Vote[LB<0 & UB>0]=0
Pos=length(Vote[Vote==1])
Neg=length(Vote[Vote==(-1)])
NS=length(Vote[Vote==0])
if (Pos > Neg & Pos > NS) VoteMaj=(+1)
if (Neg > Pos & Neg > NS) VoteMaj=(-1)
if (NS >= Neg & NS >= Pos) VoteMaj=0

#Lowest CV (MAMA)
CV=SE/abs(MU_est)
MUcv=MU_est[CV==min(CV)]
LBcv=LB[CV==min(CV)]
UBcv=UB[CV==min(CV)]
SEcv=SE[CV==min(CV)]

#Global MA (REMA)
DATAg=data.frame(Yg=Y_o, Vg=Sigma_e_o^2)
 if (NumC>0) {
   DATAg=data.frame(Yg=c(Y_c,Y_o), Vg=c(Sigma_e_c^2,Sigma_e_o^2))
 }

Mod.g=rma(Yg,Vg,data=DATAg)
MUg=Mod.g$b
LBg=Mod.g$ci.lb
UBg=Mod.g$ci.ub
SEg=Mod.g$se

#Vote counting of individual studies
LB_ind=DATAg$Yg-1.96*sqrt(DATAg$Vg)
UB_ind=DATAg$Yg+1.96*sqrt(DATAg$Vg)
Vote_ind=LB_ind
Vote_ind[LB_ind>0]=1
Vote_ind[UB_ind<0]=(-1)
Vote_ind[LB_ind<0 & UB_ind>0]=0
Pos=length(Vote_ind[Vote_ind==1])
Neg=length(Vote_ind[Vote_ind==(-1)])
NS=length(Vote_ind[Vote_ind==0])
if (Pos > Neg & Pos > NS) VoteMajInd=(+1)
if (Neg > Pos & Neg > NS) VoteMajInd=(-1)
if (NS >= Neg & NS >= Pos) VoteMajInd=0

RESULTS[k,]=c(MU2,LB2,UB2,SE2,MUg,LBg,UBg,SEg,MUcv,LBcv,UBcv,SEcv,VoteMaj,VoteMajInd)

}

#Save results
RESULTS=as.data.frame(RESULTS)
names(RESULTS)=c("MU2","LB2","UB2","SE2","MUg","LBg","UBg","SEg","MUcv","LBcv","UBcv","SEcv","VoteMaj","VoteMajInd")
print(summary(RESULTS))
nameTAB=paste("Sim", NumMA,NumS,Prop,MU,K2, ".txt")
write.table(RESULTS, file=nameTAB)
}
```

B. Proportions of correct conclusion as a function of the proportion of common data across MAs (no publication bias)

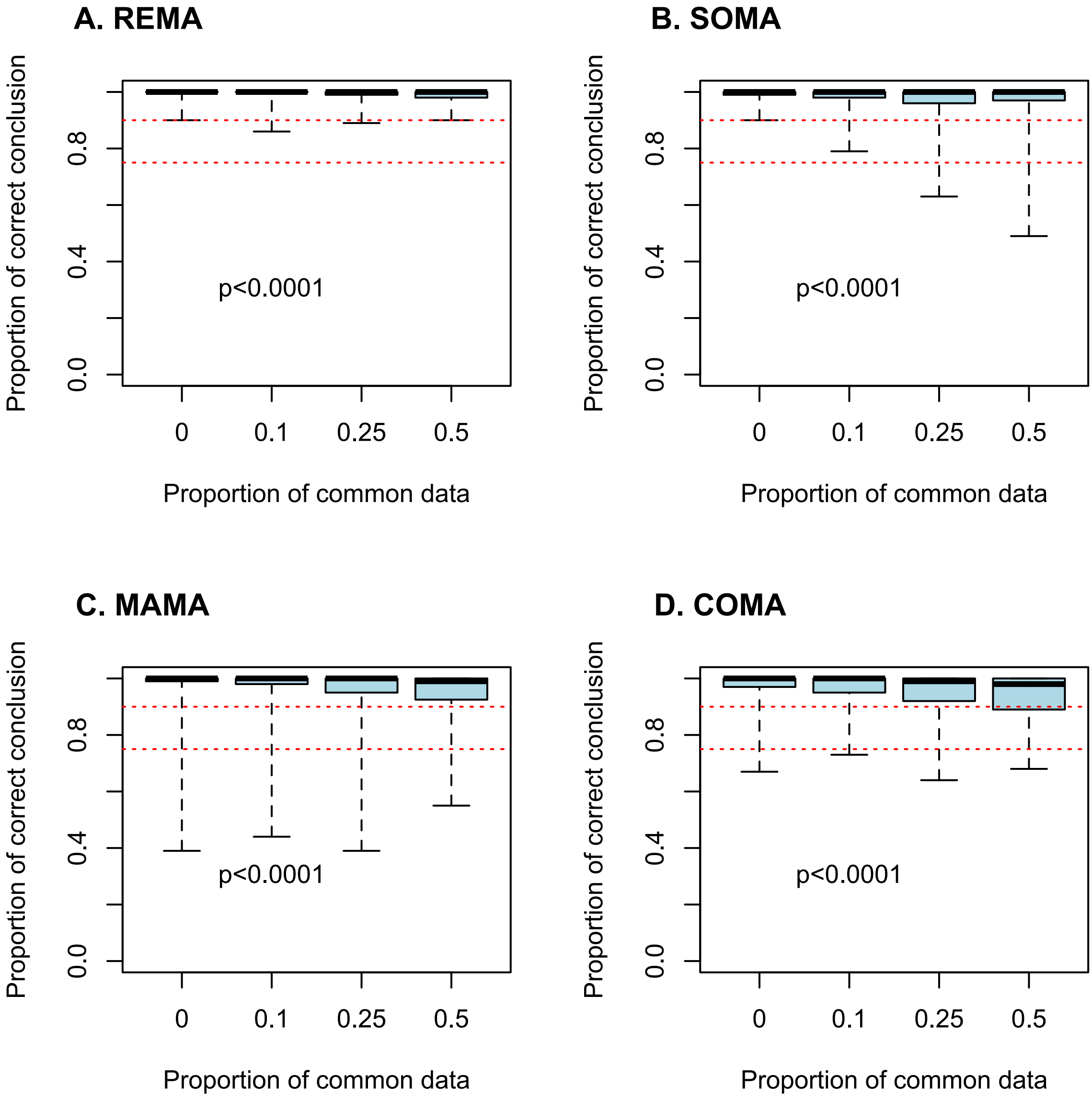

C. Results of the analysis of the factors explaining the variability of the levels of coverage of the 95% confidence intervals of the methods SOMA and MAMA, and the bias of MAMA.

```
#Analysis of the factors influencing the coverage of the CI of SOMA

Call:
glm(formula = log(CI_MA2) ~ NumMA + NumS + Prop + K2 + as.factor(MU), data = TABdec)

Deviance Residuals:
     Min        1Q     Median        3Q       Max
-0.276412  -0.049282  -0.001928   0.050526   0.193575

Coefficients:
                      Estimate    Std. Error t value Pr(>|t|)
(Intercept)           4.884e-02  1.437e-02   3.397 0.000718 ***
NumMA                 -2.424e-02 9.614e-04 -25.212  < 2e-16 ***
NumS                  1.453e-03  1.836e-04   7.914 9.59e-15 ***
Prop                  -6.543e-01 1.503e-02 -43.542  < 2e-16 ***
K2                    1.011e-02  1.377e-02   0.734 0.463272
as.factor(MU)         -0.29  2.774e-03  8.950e-03   0.310 0.756665
as.factor(MU)0        -8.481e-05  8.950e-03  -0.009 0.992442
as.factor(MU)0.22     1.486e-03  8.950e-03   0.166 0.868154
as.factor(MU)0.41     -1.025e-03  8.950e-03  -0.114 0.908894
---
Signif. codes:  0 '***' 0.001 '**' 0.01 '*' 0.05 '.' 0.1 ' ' 1

(Dispersion parameter for gaussian family taken to be 0.005766926)

Null deviance: 19.0650  on 719  degrees of freedom
Residual deviance:  4.1003  on 711  degrees of freedom
AIC: -1657.8

Number of Fisher Scoring iterations: 2

#Analysis of the factors influencing the coverage of the CI of MAMA

Call:
glm(formula = log(CI_MAcv) ~ NumMA + NumS + Prop + K2 + as.factor(MU),
   data = TABdec)

Deviance Residuals:
    Min       1Q    Median       3Q      Max
-0.41726  -0.03579   0.00162   0.04335   0.21468

Coefficients:
                      Estimate       Std. Error t value Pr(>|t|)
(Intercept)           -0.0770899  0.0143194  -5.384 9.93e-08 ***
NumMA                 -0.0281364  0.0009577 -29.379  < 2e-16 ***
NumS                  0.0019440   0.0001829  10.626  < 2e-16 ***
Prop                  0.1254793   0.0149703   8.382 2.78e-16 ***
K2                    0.0059634   0.0137209   0.435    0.664
as.factor(MU)-0.29    0.0055994   0.0089157   0.628    0.530
as.factor(MU)0        -0.2226720  0.0089157 -24.975  < 2e-16 ***
as.factor(MU)0.22     -0.0090460  0.0089157  -1.015    0.311
as.factor(MU)0.41     0.0008753   0.0089157   0.098    0.922
---
Signif. codes:  0 '***' 0.001 '**' 0.01 '*' 0.05 '.' 0.1 ' ' 1

(Dispersion parameter for gaussian family taken to be 0.005723213)

   Null deviance: 15.7538  on 719  degrees of freedom
Residual deviance:  4.0692  on 711  degrees of freedom
AIC: -1663.3

Number of Fisher Scoring iterations: 2

#Analysis of the factors influencing the bias of MAMA
```

```
Call:
glm(formula = bMAcv ~ NumMA + NumS + Prop + K2 + as.factor(MU), data = TABdec)

Deviance Residuals:
     Min        1Q    Median        3Q       Max
-0.163637  -0.016581   0.000319   0.015632   0.118815

Coefficients:
                     Estimate  Std. Error t value Pr(>|t|)
(Intercept)          -1.521e-01  6.418e-03 -23.700  < 2e-16 ***
NumMA                -9.589e-04  4.292e-04  -2.234  0.02579 *
NumS                  2.493e-04  8.199e-05   3.040  0.00245 **
Prop                  8.517e-03  6.710e-03   1.269  0.20469
K2                   -6.890e-03  6.150e-03  -1.120  0.26294
as.factor(MU)-0.29    8.929e-02  3.996e-03  22.345  < 2e-16 ***
as.factor(MU)0        1.549e-01  3.996e-03  38.757  < 2e-16 ***
as.factor(MU)0.22     2.065e-01  3.996e-03  51.680  < 2e-16 ***
as.factor(MU)0.41     2.471e-01  3.996e-03  61.826  < 2e-16 ***
---
Signif. codes:  0 ‘***’ 0.001 ‘**’ 0.01 ‘*’ 0.05 ‘.’ 0.1 ‘ ’ 1

(Dispersion parameter for gaussian family taken to be 0.001149633)

Null deviance: 6.34862  on 719  degrees of freedom
Residual deviance: 0.81739  on 711  degrees of freedom
AIC: -2819

Number of Fisher Scoring iterations: 2
```

D. Examples of samples of individual effect sizes (*N*=50) generated with and without publication bias. In each graphic, the black points are the 50 simulated individual effect sizes, the blue line is the true mean effect size value (here, -0.69), the red line indicates the mean effect size estimated by a MA of the 50 individual effect sizes, the white points are the number of missing studies estimated with the trim and fill method (the white point not used by the MA). Three types of publications bias were considered (see main text)

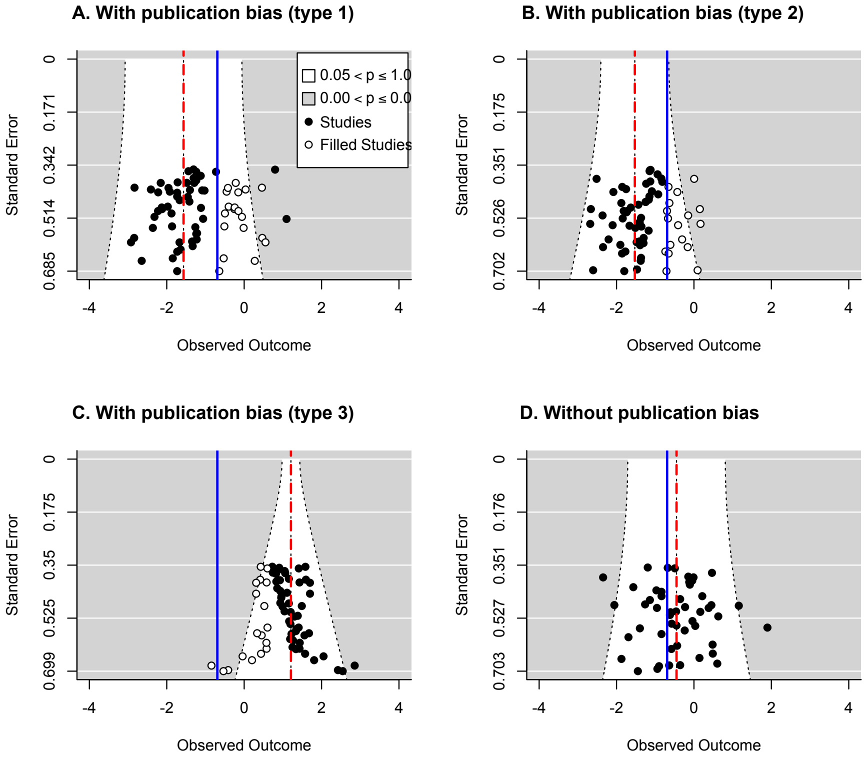

E. Impact of publication bias for *K*=5 first-order MAs. The numbers at the top of the figures indicate the true mean effect sizes considered. The computations were performed assuming a true mean effect size equal to -0.29 (loss of -25%, left), -0.69 (loss of -50%, middle), or zero (no effect, right).

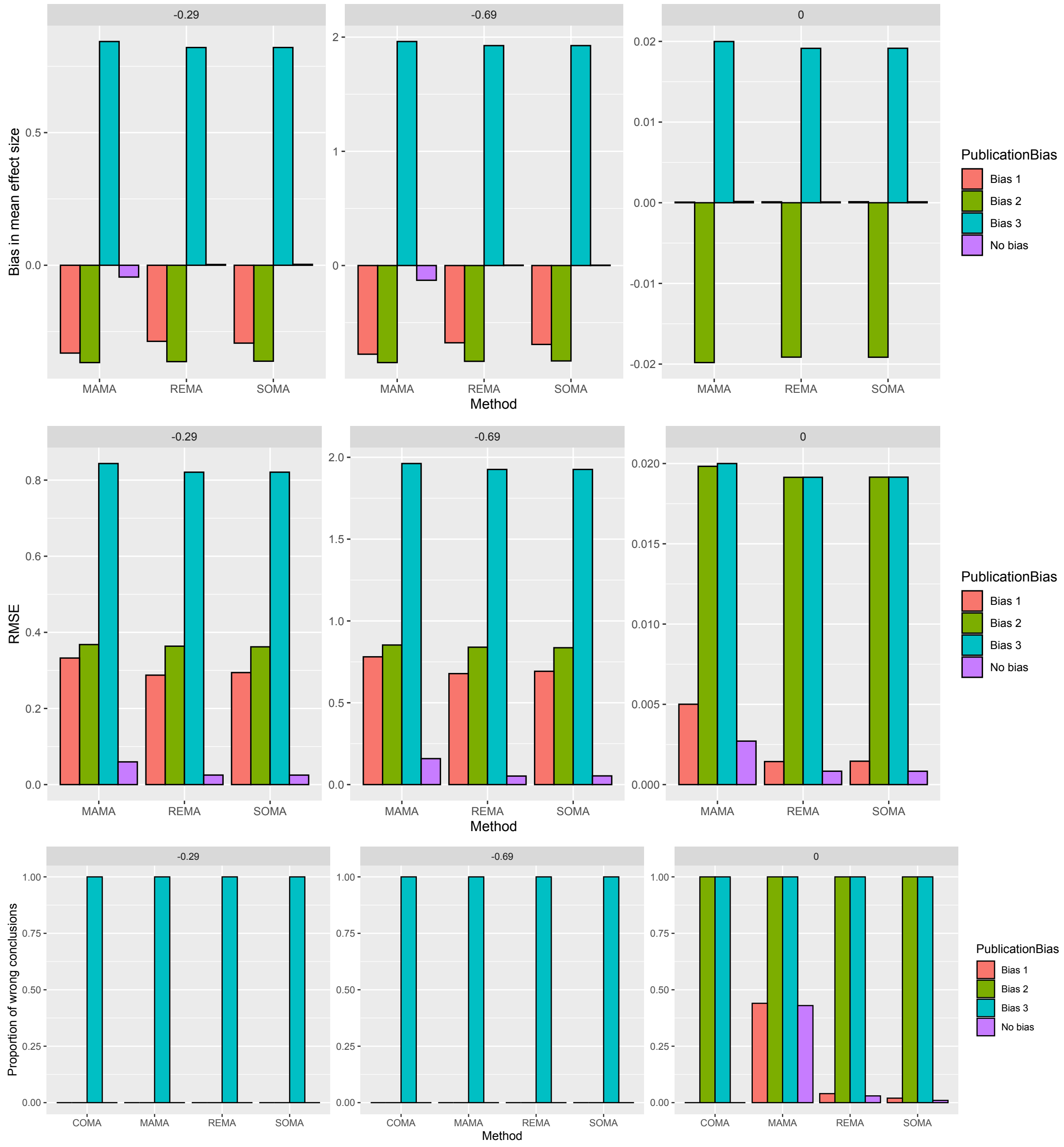

F. Impact of publication bias for K=3 first-order MAs. The numbers at the top of the figures indicate the true mean effect sizes considered. The computations were performed assuming a true mean effect size equal to -0.29 (loss of -25%, left), -0.69 (loss of -50%, middle), or zero (no effect, right).

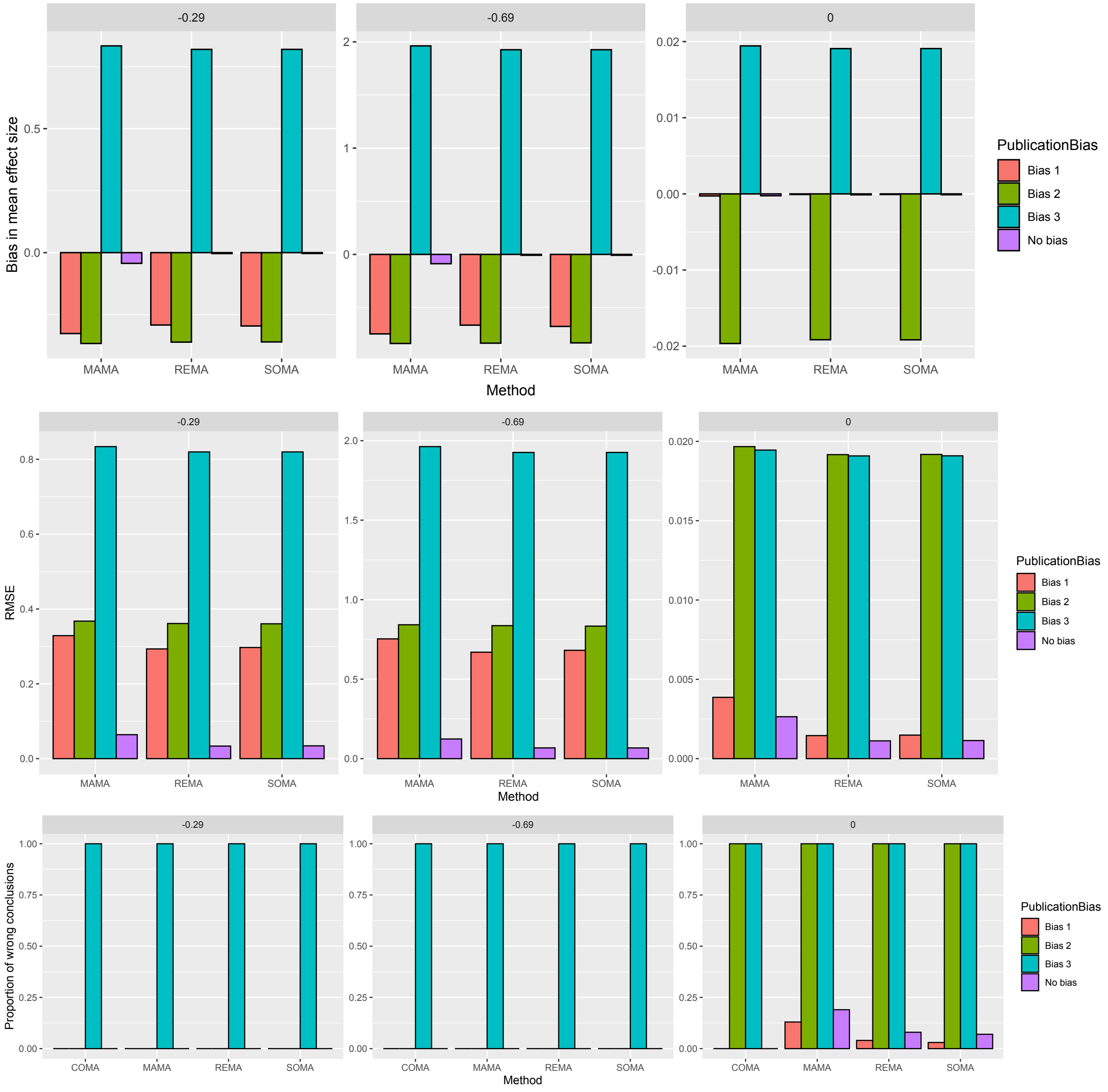

Supplement: Supplementary file 1 — Additional file 1. Code used to generate simulated data and additional results. [file 13750_2023_309_MOESM1_ESM.pdf]
